# Supplementary material for: Worldviews and the role of social values that underlie them
Source: PLoS One. 2023 Jul 26;18(7):e0288451. doi: 10.1371/journal.pone.0288451 (PMC10370735; doi:10.1371/journal.pone.0288451)
Supplement: Attachment — Submitted filename: review PONE-D-23-05362.pdf [file pone.0288451.s002.pdf]

Review of "Worldviews and the role of social values that underlie them"

I found this article very interesting, theoretically insightful and with a potential for theoretical contribution in the field. I highlight some aspects, generally minor ones, that may improve some aspects of the manuscript that are not very clear. I present my comments divided in to the different sections of the manuscript.

ABSTRACT

It is not clear in the abstract what is the main objective of the manuscript. Is it to propose a typology of worldviews (I assume that this has already been proposed/published elsewhere but this is unclear here)? Is it to identify values? Is to theoretically suggest a relationship between values and worldviews and support it through data? Having read the paper, it is clearly the third one, but I think this should be reflected in the abstract. Overall, I think the abstract needs a clearer organisation of aims and theoretical background.

INTRODUCTION

I think that the introduction also needs a clearer articulation of the argument and its importance for the literature.

Regarding the terms used: what is the difference between belief systems, ideologies and worldviews? I see that these are explained further down in separate section, but in order for the introduction to demonstrate clearly the argument, there needs to be a shorter discussion of their distinction? Judging from this paragraph, it seems that the focus of this paper is on belief systems (so is the focus of this paragraph as well) but then further down, the concept of worldviews becomes central.

This is linked to my next comment regarding the relationship between social values and belief sets that have just been introduced and the ways they relate to worldviews.

Is this typology already presented/published in the literature? I see that you explain that later, but I do believe that the intro needs to stand on its own to demonstrate the argument

Most importantly: Why do we need such a theoretical account and empirical demonstration of their relationship? What is the importance of this?

SOCIAL VALUES

I guess belief structures are belief sets? maybe avoid using different terms for the same concepts to avoid confusion.

HYPOTHESIZED RELATIONSHIP BETWEEN VALUES AND WORLDVIEWS

I appreciate the theoretical depth and interesting theoretical insights provided in this section. I think that it needs to become clearer in the text whether this theoretical relationship is something that you wish to suggest and advance in this manuscript or whether it is something already achieved/presented in previous publications.

Overall, in the literature review of the manuscript I feel that main argumentative thread is missing and that the sections could benefit from clearer linking to each other.

I think that the need to identify the link between values and worldviews should become more prominently argued in the text.

DATA ANALYSES

It is not clear why cultural differences were measured in terms of continents where participants belonged. I think this point needs some justification.

RESULTS

The analytical choices and the procedures followed seem to be suited to respond to the questions regarding the relationship between worldviews and values (Correlational analysis) and whether worldviews predict values (regression analyses). The findings are interesting and robust. It is particularly interesting to see how different worldviews may converge and diverge in their relationship with certain values.

DISCUSSION

The interpretation of the findings in the first section of the discussion is deep, interesting and meticulous.

CONCLUSION AND FUTURE DIRECTIONS

Regarding the claim made: “Specifically, the empirical relationship between worldviews and values facilitates the understanding of how individuals may come together and agree to support a cause or a course of action despite clear and widespread intra-group differences”. This is very interesting-I think it should come earlier, as part of the argument of why we need to examine the relationship between the two. However, the distance between values and actions should also be acknowledged here.

In the same section, in the last two paragraphs I appreciate the suggestions for further research that engage with both intra-individual and contextual aspects in relation to adoption of worldviews. However, I would expect these to appear more in the first demonstration/publication of the typology rather than in this manuscript, where the relationship between values and worldviews is discussed. For this reason I urge the authors to delve more into the first point of discussion and expand on the implications of looking at this relationship they examined in this manuscript.
