# Supplementary material for: Worldviews and the role of social values that underlie them
Source: PLoS One. 2023 Jul 26;18(7):e0288451. doi: 10.1371/journal.pone.0288451 (PMC10370735; doi:10.1371/journal.pone.0288451)
Supplement: Attachment — Submitted filename: Response Letter.docx [file pone.0288451.s003.docx]

**Response Letter**

**Manuscript ID:** PONE-D-23-05362

**Manuscript title:** Worldviews and the role of social value that underlie them

Dear Editor,

We hope this letter finds you well. We are writing to express our heartfelt gratitude for the time, expertise, and invaluable recommendations provided during the review of our manuscript. The reviewers’ expertise and keen observations have undoubtedly contributed to a significant improvement in our work, and we are sincerely appreciative of it. The suggestions for further exploration of certain aspects have been noted and addressed as follows:

**Reviewer #1:**

- “This was a very interesting paper that contributes to the theorisation and empirical study of socio-cognitive mechanisms of worldviews/values. It was wonderful to see that you try to advance a more situational understanding of the concept, which is lacking in the field. I have some minor comments that I hope can be helpful to improver your wonderful work.”

Response: Thank you for this. We are sincerely grateful for your acknowledgement on the significance of our findings and their potential to advance knowledge in this field.

- “Even though you clarify the similarities between worldviews and the other concepts are, it is not very clear what are the differences between them and how the idea of the worldview is a better choice. Is it possible to explain a bit more?”

Response: The way in which worldviews differ from the other construct is in terms of how it is measured. Through vignettes, worldviews offer a holistic formulation that we believe add up to more than the sum of a sequence of Likert scales that are added up to form a composite. We have now provided more detail of this in page 3 in the Worldviews section.

- “You are highlighting the importance of situational demands in the conceptualisation and operationalisation of worldviews. Yet it is not entirely clear how this is reflected in the operationalisation of the construct i.e. in the vignettes. For example, who are these ‘others’ in the scenarios, who constitutes an in-group/out-group can vary across situations and topics/projects of interest. You introduce this idea towards the conclusion but it is important to clarify what situational aspects (if any) the vignettes are taking into account. Another point is that you are drawing on Schwartz’s values which are quite abstract and do not take into account the importance of situations and contexts. Can you please explain address this?”

Response: The participant is free to draw upon any situation and context because we belief that, despite the differences in situation and contexts that participants draw upon, the psychological outlook captured by the worldviews remains the same. We have now explained this in page 3 in the Worldviews section.

- “Can you please explain a bit more why you chose for your study Schwartz’s values as opposed to the other constructs you review in your introduction?”

Response: Unlike other types of belief systems (e.g., political and religious), values are more abstract than worldviews. For this reason, they offer an opportunity to identify the core elements or motivational constituents that form a particular worldview. We have added a clarification for this point in page 5 at the start of the section “The Hypothesized Relationship Between Worldviews and Values”.

- “In the sampling section, can you please provide some more information about the educational background of the participants? Did you also control for that in your analyses? If not, it's ok. Can you just please explain why.”

Response: Unfortunately, we did not control for educational background of the participants. We have added a section dedicated to this in page 20 towards the end of the Conclusion section.

- “In the results section, some of the correlations are quite weak. Can you please comment on that and what implications this has for your theorisations and findings?”

Response: This is certainly a legitimate point of consideration. Our findings support the notion that worldviews are better suited for explaining some proportion of variance rather than merely establishing correlations. We have provided a comment on this in page 17 of the Discussion section.

- “In the results section, why did you choose to perform the analyses with the higher order values eg self-transcendence and not the individual ones e.g. benevolence, universalism ? There has been some evidence that goes against Schwartz’s value relationships. Can you explain your rationale?”

Response: Schwartz’s higher order values are similar to Trandis’ notion of individualism-collectivism dimensions, which have been linked to cultural worldviews. Being that, at a cultural-level, a link has been established between values and worldviews, we wanted to investigate whether the same can be established at the individual level. For this reason, we opted to explore the potential link between Schwartz’s higher order values (which are akin to Triandis) and worldviews. A better explanation for this can be found in page 5 in the section “The Hypothesized Relationship Between Worldviews and Values”.

- “In terms of further research and reflections, the vignettes seem to capture rather secular and anthropocentric worldviews. What about relationships to more than human worlds eg nature, spirits, but also orientations to time (role of temporality- how we understand history links to our worldviews maybe Lola Olufemi could be an inspiration?). Another question is: Does a worldview include what is or also what could be different? Perhaps it would be interesting to engage with questions about speculation and world-making e.g. Power, S. A., Zittoun, T., Akkerman, S., Wagoner, B., Cabra, M., Cornish, F., Hawlina, H., Heasman, B., Mahendran, K., Psaltis, C., Rajala, A., Veale, A., & Gillespie, A. (2023). Social Psychology of and for World-Making. Personality and Social Psychology Review, 0(0). https://doi.org/10.1177/10888683221145756”

Response: We would expect these concerns (i.e., nature, spirits, temporal factors, etc.) to also be interpreted though the “worldviews lens”. This has now been addressed in page 3 towards the end of the Worldviews section in which we also added further clarification on our conceptualisation of worldviews as well as a comment regarding world-making.

- “In your conclusion, you argue about the role of situations and you bring the example of poor versus rich people. You argue ‘poverty may predispose individuals towards a Survivor worldview whilst riches might predispose others towards a Reward worldview’ Actually this is an erroneous belief. There is a lot of evidence to the contrary. You can refer to literature on development eg Robert Chambers, Arturo Escobar”

Response: This is a noteworthy observation. We have removed this example and instead discussed the potential implications of other adverse life events on shifting one’s worldview. Kindly find this in page 20 towards the end of the Conclusion section.

**Reviewer #2:**

- “I found this article very interesting, theoretically insightful and with a potential for theoretical contribution in the field. I highlight some aspects, generally minor ones, that may improve some aspects of the manuscript that are not very clear. I present my comments divided in to the different sections of the manuscript.”

Response: Thank you for this. We are sincerely grateful for your words and on your positive assessment of our potential contributions in the field.

- “It is not clear in the abstract what is the main objective of the manuscript. Is it to propose a typology of worldviews (I assume that this has already been proposed/published elsewhere but this is unclear here)? Is it to identify values? Is to theoretically suggest a relationship between values and worldviews and support it through data? Having read the paper, it is clearly the third one, but I think this should be reflected in the abstract. Overall, I think the abstract needs a clearer organisation of aims and theoretical background.”

Response: Thank you for this. We have refined the language and structure of the abstract to ensure that the main point of our research is clearer. We have also dedicated more detail to the results section in the abstract to underscore the investigation of worldviews and values within our study.

- “I think that the introduction also needs a clearer articulation of the argument and its importance for the literature.

Regarding the terms used: what is the difference between belief systems, ideologies and worldviews? I see that these are explained further down in separate section, but in order for the introduction to demonstrate clearly the argument, there needs to be a shorter discussion of their distinction? Judging from this paragraph, it seems that the focus of this paper is on belief systems (so is the focus of this paragraph as well) but then further down, the concept of worldviews becomes central.

This is linked to my next comment regarding the relationship between social values and belief sets that have just been introduced and the ways they relate to worldviews.

Is this typology already presented/published in the literature? I see that you explain that later, but I do believe that the intro needs to stand on its own to demonstrate the argument

Most importantly: Why do we need such a theoretical account and empirical demonstration of their relationship? What is the importance of this?”

Response: Worldviews and ideologies are a types of belief systems. To make this clearer we have added a concise overview of worldviews in the introduction, highlighting their significance within the context of our research. We have also included a justification for the investigation of the link between worldviews and values. Kindly find this in page 1 and 2 in the Introduction.

- “I guess belief structures are belief sets? maybe avoid using different terms for the same concepts to avoid confusion.”

Response: Agreed, we have changed all such terms to the term “belief system”.

- “I appreciate the theoretical depth and interesting theoretical insights provided in this section. I think that it needs to become clearer in the text whether this theoretical relationship is something that you wish to suggest and advance in this manuscript or whether it is something already achieved/presented in previous publications.

Overall, in the literature review of the manuscript I feel that main argumentative thread is missing and that the sections could benefit from clearer linking to each other.

I think that the need to identify the link between values and worldviews should become more prominently argued in the text.”

Response: Thank you for this. We have shifted the sections around such that now the section on worldviews precedes the one on social values. We have also added more detail, to the section linking the two together (the hypothesized relationship between worldviews and values), providing more rationale for our research, emphasizing why we chose to investigate values rather than other constructs. Most of these changes can be found on page 3, towards the end of the Worldviews section, and on page 5, at the start of the section titled “The hypothesized relationship between worldviews and values”.

- “It is not clear why cultural differences were measured in terms of continents where participants belonged. I think this point needs some justification.”

Response: Agreed. We wanted to control for cultural differences in some way. However, since participants resided in a disproportionate variety of countries, these were grouped together in terms of continent. We this detail on page 13 of the data analyses section.

- “The analytical choices and the procedures followed seem to be suited to respond to the questions regarding the relationship between worldviews and values (Correlational analysis) and whether worldviews predict values (regression analyses). The findings are interesting and robust. It is particularly interesting to see how different worldviews may converge and diverge in their relationship with certain values.”

“The interpretation of the findings in the first section of the discussion is deep, interesting and meticulous.”

Response: We are truly thankful for your positive assessment of our analysis and discussion, it reaffirms our efforts to ensure a robust and reliable study.

- “Regarding the claim made: “Specifically, the empirical relationship between worldviews and values facilitates the understanding of how individuals may come together and agree to support a cause or a course of action despite clear and widespread intra-group differences”. This is very interesting-I think it should come earlier, as part of the argument of why we need to examine the relationship between the two. However, the distance between values and actions should also be acknowledged here.

In the same section, in the last two paragraphs I appreciate the suggestions for further research that engage with both intra-individual and contextual aspects in relation to adoption of worldviews. However, I would expect these to appear more in the first demonstration/publication of the typology rather than in this manuscript, where the relationship between values and worldviews is discussed. For this reason I urge the authors to delve more into the first point of discussion and expand on the implications of looking at this relationship they examined in this manuscript.”

Response: Agreed. Worldviews have great potential to function as coalitional mechanisms amongst seemingly disparate groups. We have taken your suggestion and included some points from this claim as part of our justification for investigating the link between worldviews and values. Kindly find this in page 2 towards the middle part of the Introduction.

**Editorial office:**

- “Please provide additional details regarding participant consent. In the ethics statement in the Methods and online submission information, please ensure that you have specified what type you obtained (for instance, written or verbal, and if verbal, how it was documented and witnessed). If your study included minors, state whether you obtained consent from parents or guardians. If the need for consent was waived by the ethics committee, please include this information.”

Response: Additional details pertaining to participant consent has been included in page 10 in the subsection titled “Participants and procedure” in the Methodology section.

- “Please include your full ethics statement in the ‘Methods’ section of your manuscript file. In your statement, please include the full name of the IRB or ethics committee who approved or waived your study, as well as whether or not you obtained informed written or verbal consent. If consent was waived for your study, please include this information in your statement as well.”

Response: An ethics statements has been included in page 10 in the subsection titled “Participants and procedure” in the Methodology section.

Once again, we would like to express our gratitude for the reviewers’ valuable feedback and constructive criticism on our manuscript. Their thoughtful comments have been instrumental in shaping our research and manuscript, improving its overall quality. Thank you also for your time, and thank you for the opportunity to revise our manuscript.
